# MicroRNAs regulating cluster of differentiation 46 (*CD46*) in cardioembolic and non-cardioembolic stroke

**DOI:** 10.1371/journal.pone.0172131

**Published:** 2017-02-15

**Authors:** Jun Rong Tan, Kay Sin Tan, Fung Lin Yong, Arunmozhiarasi Armugam, Chee Woon Wang, Kandiah Jeyaseelan, Peter Tsun-Hon Wong

**Affiliations:** 1 Department of Biochemistry, Yong Loo Lin School of Medicine, National University of Singapore, Singapore; 2 Department of Medicine, Faculty of Medicine, University Malaya, Kuala Lumpur, Malaysia; 3 Department of Biochemistry, Faculty of Medicine, MAHSA University, Kuala Lumpur, Malaysia; 4 Department of Anatomy and Developmental Biology, School of Biomedical Sciences, Faculty of Medicine, Nursing and Health Sciences, Monash University, Clayton, Victoria, Australia; 5 Department of Pharmacology, Yong Loo Lin School of Medicine, National University of Singapore, MD3, Singapore; Institut d'Investigacions Biomediques de Barcelona, SPAIN

## Abstract

Ischemic stroke is a major cause of mortality and morbidity globally. Among the ischemic stroke subtypes, cardioembolic stroke is with poor functional outcome (Modified Rankin score ≥ 2). Early diagnosis of cardioembolic stroke will prove beneficial. This study examined the microRNAs targeting cluster of differentiation 46 (CD46), a potential biomarker for cardioembolic stroke. CD46 mRNA level was shown to be differentially expressed (*p* < 0.001) between cardioembolic stroke (median = 1.32) and non-cardioembolic stroke subtypes (large artery stroke median = 5.05; small vessel stroke median = 6.45). Bioinformatic search showed that miR-19a, -20a, -185 and -374b were found to target CD46 mRNA and further verified by luciferase reporter assay. The levels of miRNAs targeting CD46 were significantly reduced (*p* < 0.05) in non-cardioembolic stroke patients (large artery stroke median: miR-19a = 0.63, miR-20a = 0.42, miR-185 = 0.32, miR-374b = 0.27; small artery stroke median: miR-19a = 0.07, miR-20a = 0.06, miR-185 = 0.07, miR-374b = 0.05) as compared to cardioembolic stroke patients (median: miR-19a = 2.69, miR-20a = 1.36, miR-185 = 1.05, miR-374b = 1.23). ROC curve showed that the miRNAs could distinguish cardioembolic stroke from non-cardioembolic stroke with better AUC value as compared to CD46. Endogenous expression of CD46 in Human Umbilical Vein Endothelial Cells (HUVECs) were found to be regulated by miR-19a and miR-20a. Thus implicating that miR-19a and -20a may play a role in pathogenesis of cardioembolic stroke, possibly *via* the endothelial cells.

## Introduction

Ischemic stroke is one of the leading causes of death and disabilities worldwide. It accounts for 80% of all reported stroke cases [1–3]. According to Trial of Org 10172 in Acute Stroke Treatment (TOAST) classification [4], among the different ischemic stroke subtypes, cardioembolic stroke, a type of ischemic stroke characterized by the blood clot originating from a cardioembolic source, is associated with poor functional outcome (based on Modified Rankin score) in patients [5]. However, the current challenge for cardioembolic stroke diagnosis is the lack of an accurate diagnosis to segregate cardioembolic stroke cases from large artery stroke cases (which is another common ischemic stroke subtype). Hence, identification of specific biomarkers for cardioembolic stroke will prove beneficial.

Previous reports had highlighted a group of mRNAs which can potentially classify ischemic stroke patients into their various stroke subtypes [6], [7]. Among these mRNAs, there was an inhibitor of complement cascade, cluster of differentiation 46 (*CD46*) [8], which could distinguish cardioembolic stroke from non-cardioembolic stroke subtype [6], [7]. Components of complement cascade that had been found to be increased in ischemic stroke patients, have also been observed to be associated with stroke outcome [9–11]. This denotes that complement cascade is deregulated during ischemic stroke and hence can be a potential biomarker for ischemic stroke. Thus, it would be beneficial to investigate how the differential expression of *CD46* contributes to the pathogenesis of the different stroke subtypes.

As the expression pattern of microRNAs (miRNAs) present in blood changes in response to different disease states, we studied them to obtain information on the molecular mechanism of cardioembolic stroke pathogenesis involving *CD46*. miRNAs are a class of short (18–22 nucleotides), endogenously expressed, non-coding RNAs that act as riboregulators of gene expression [12], [13]. Typically, miRNAs down-regulate gene expression of target mRNA through the process of RNA interference (RNAi) [13]. A single miRNA can target multiple genes and it can also have an overall effect on certain pathways. Hence, looking into miRNAs targeting *CD46* can potentially uncover the differential pathway regulation between cardioembolic and non-cardioembolic stroke. Thus, this study was aimed to identify the miRNAs targeting *CD46* as they may serve as potential biomarkers and to uncover the mechanism behind differential expression of *CD46* between cardioembolic and non-cardioembolic stroke.

## Materials and methods

### Patient recruitment

This study was approved by the Medical Ethics Committee of the University Malaya Medical Centre (UMMC), Kuala Lumpur, Malaysia (Ref. No: 607.20) and the Institutional Review Board (IRB) of the National University of Singapore (NUS; NUS-IRB Ref Code: 08–381; Approval: NUS-676), and was carried out in accordance with the Declaration of Helsinki (2008). Written informed consent was obtained from patients and was carried out with the approval of the ethics committee and IRB. The patients were recruited from University Malaya Medical Centre (UMMC), Kuala Lumpur, Malaysia. The patients were admitted into UMMC via the Neurology service and Accident and Emergency (A&E) department. Ischemic stroke in patients were diagnosed through either magnetic resonance imaging (MRI) or computed tomography (CT) scan. Blood samples were collected from patients within 24 hours following admission. Peripheral blood was collected by venepuncture into BD Vacutainer with no additive (BD, Franklin Lakes, NJ, US; Cat# 366703) and was immediately aliquoted into Eppendorf tubes containing RNA later (Ambion, Life Technology, Carlsbad, CA, US) according to manufacturer’s protocol. These samples were stored at -80°C until required.

39 patients and 18 healthy controls were recruited for this study. Patients were classified according to the Trial of Org 10172 in Acute Stroke Treatment (TOAST) classification [4] (Table 1). The outcomes of the patients were assessed using the modified Rankin scale (mRS) [5] and dichotomized into good (mRS < 2) and poor outcome (mRS ≥ 2; Table 1).

**Table 1:** Patients’ demography.

	Control (n = 18)	CE stroke (n = 13)	Non-CE stroke (n = 26)	*p-value* (CE vs Non-CE)
Age (Mean ± SD)	42.87 ± 12.42	67.50 ± 16.13	62.84 ± 11.61	*0*.*19*
Female, n (%)	6(33.33%)	5 (41.67%)	10 (38.46%)	*0*.*27*
Dyslipidaemia, n (%)	NA	9 (75.00%)	19 (73.08%)	*0*.*30*
Diabetes mellitus, n (%)	NA	5 (41.67%)	14 (53.85%)	*0*.*21*
Hypertension, n (%)	NA	5 (41.67%)	20 (76.92%)	*0*.*03*
Ischemic heart disease, n (%)	NA	4 (33.33%)	6 (23.08%)	*0*.*24*
Atrial fibrillation, n (%)	NA	10 (83.33%)	0 (0.00%)	*<0*.*001*
Previous stroke, n (%)	NA	3 (25.00%)	3 (11.54%)	*0*.*20*
Smoking, n (%)	NA	0 (0.00%)	5 (19.23%)	*0*.*13*
Alcohol, n (%)	NA	0 (0.00%)	1 (3.84%)	*0*.*68*
Modified Rankin Scale < 2, n (%)	NA	1 (8.33%)	6 (23.07%)	*0*.*21*

The patients were classified according to cardioembolic stroke and non-cardioembolic stroke (large artery stroke and small vessel stroke). The various parameters were tested using Mann-Whitney U test. CE: cardioembolic. Non-CE: Non-cardioembolic.

### Transfection of miRNAs in HUVECs

Human umbilical vein endothelial cells (HUVEC, CRL-1730; ATCC, US) were cultured using Dulbecco’s Modified Eagle Medium (DMEM; Gibco, Carlsbad, CA, US) supplemented with 10% fetal bovine serum (Gibco, Carlsbad, CA, US) and 1% penicillin-streptomycin (Gibco, Carlsbad, CA, US) and maintained at 37°C in 5% carbon dioxide. HUVECs were seeded at density of 3 X 10^4^ cells in each well of 24 well plates. Human anti-miRNAs and miRNA mimics were transfected at final concentration of 30 nM in 50 μl of Opti-MEM (Gibco, Carlsbad, CA, US) complexed with 1 μl of NeoFx in 50 μl of Opti-MEM (Gibco, Carlsbad, CA, US) per well. Cells were cultured at 37°C in 5% carbon dioxide for 48 hours.

### RNA isolation

Total RNAs (+miRNAs) were isolated from blood using Ambion Ribopure blood extraction kit (Ambion, Life Technology, Carlsbad, CA, US) according to manufacturer’s protocol. Total RNAs (including miRNAs) were isolated from cells using TRIzol reagent (Invitrogen, Carlsbad, CA, US) according to manufacturer’s protocol. The concentration and integrity of total RNAs were determined by the Nano-Drop ND-1000 Spectrophotometry (NanoDrop Tech, Wilmington, DE, US) and denaturing agarose gel electrophoresis.

### Cloning of *CD46* 3’UTR fragment

Gene specific primers were used to amplify the region of *CD46* 3’UTR containing the seed regions of the miRNAs (forward primer, 5’- GACAGCCATAACAGGAGTGC– 3’; reverse primer, 5’–ACTTCCAGAGAAAACTGGTC– 3’). The PCR product was cloned into pMIR-REPORT Luciferase vector (Promega, Madison, WI, US) at the *Spe*I and *Hind*III multi-cloning sites.

### Luciferase assay

Luciferase assay was performed according to Sepramaniam et al [14]. HeLa cells (CCL-2; ATCC, USA) were cultured using Dulbecco’s Modified Eagle Medium (DMEM; Gibco, Carlsbad, CA, US) supplemented with 10% fetal bovine serum (Gibco, Carlsbad, CA, US) and 1% penicillin-streptomycin (Gibco, Carlsbad, CA, US) and maintained at 37°C in 5% carbon dioxide. Cells were transfected with 50nM anti-miRNAs or miRNA mimics for 3 hours followed by 100 ng/well pMIR-REPORT Luciferase vector for 3 hours as previously shown by Sepramaniam et al [14] and Kaur et al [15]. The cells were lysed 48 h later for measurement of luciferase activity. Dual luciferase assay (Promega, Madison, WI, US) was used to quantitate the effects of anti- or pre-miRNA interaction with *CD46* 3’UTR. The assay was performed according to the manufacturer’s protocol. In all experiments, transfection efficiencies were normalized to those of cells co-transfected with the *Renilla luciferase* expressing vector (pRL-CMV; Promega, Madison, WI, US) at 10 ng/well.

### Reverse transcription and real-time qualitative polymerase chain reaction (qPCR)

Reverse transcription followed by real-time qualitative PCR were carried out according to Kaur et al. [15] For gene quantification, reverse transcription was performed using TaqMan Reverse Transcription kit (Applied Biosystems, Foster City, CA, US) according to manufacturer’s protocol. Quantification of *CD46* mRNA was performed using SYBR Green assay (Applied Biosystems, Foster City, CA, US) and *GAPDH* was used as an endogenous control [15] for normalization (*CD46* forward primer, 5’-GGTGTTGCTGCTGTACTCCTTCT-3’; *CD46* reverse primer, 5’-CCAATGAGCTCCATAGCTTCAA-3’; *GAPDH* forward primer, 5’-AACAGCGACACCCATCCTC-3’; *GAPDH* reverse primer, 5’-CATACCAGGAAATGAGCTTGACAA-3’)

For microRNA quantification, reverse transcription was performed using TaqMan MicroRNA Reverse Transcription kit (Applied Biosystems, Foster City, CA, US) with microRNA-specific stem-loop primers. The microRNA expression was normalized to endogenous control *GAPDH* [15]. Subsequently, miRNA quantitation was performed using TaqMan chemistry. All reactions were conducted on Applied Biosystems 7900HT Fast Real-time PCR system (Applied Biosystems, Foster City, CA, US).

### Protein gel and Western blot analysis

Protein was obtained from the phenol phase of the RNA extraction reaction (Invitrogen, Carlsbad, CA, US) and quantified using Bio-Rad protein assay (Bio-Rad, Hercules, CA, US). 40 μg of total protein was resolved using Bolt 4–12% Bis-Tris plus gel (Novex, Life Technologies, Grand Island, NY, US) and transferred using the iBlot gel transfer system (Novex, Life Technologies, Grand Island, NY, US). The membranes were probed using the iBind Flex Western Device (Thermo Fisher Scientific, Waltham, MA, US) according to manufacturer’s protocol, using primary antibodies for CD46 (1:1000; Abcam, Cambridge, UK; Cat# ab789), primary antibodies for β-Actin (1:5000; Bio-Rad, Hercules, CA, USA; Cat# MCA5775GA) and secondary antibodies (1:5000; horseradish peroxidase-conjugated goat anti-mouse; Bio-Rad, Hercules, CA, USA; Cat# 1721011). The proteins on the membranes were visualized *via* enhanced chemiluminescence (SuperSignal West; Thermo Fisher Scientific, Waltham, MA, USA) and exposed to hyperfilm (Amersham hyperfilm, GE healthcare lifescience, Little Chalfont, UK). The labelling intensities of the bands were quantitated using ImageJ software (National Institutes of Health, Bethesda, MD, US) [16].

### Immunocytochemistry

Immunocytochemistry was performed on HUVECs treated with anti-miRNAs and miRNA mimics. The cells were fixed with 4% formaldehyde in phosphate-buffered saline for 20 min, permeabilized with 0.1% Triton X-100 in PBS for 30 min, and blocked with 5% FBS in PBST for 30 min. CD46 was probed using primary antibodies for CD46 (1:100; Abcam, Cambridge, UK; Cat# ab789) and Texas Red conjugated secondary antibodies (1:200; Abcam, Cambridge, UK; Cat# ab7066). 0.1 μg/mL Hoechst 33342 (Biotium, Foster City, CA, USA) was used for visualising the nucleus. The images were viewed and analysed using LSM710 confocal imaging software (Carl Zeiss AG, Oberkochen, Germany).

### *In silico* prediction

Bioinformatics prediction of miRNAs targeting CD46 was performed using available databases online: Targetscan version 6.2 (www.targetscan.org) [17–20], miRanda August 2010 release (www.microRNA.org) [21–24], microcosm version 5 (www.ebi.ac.uk/enright-srv/microcosm/htdocs/targets/v5/) [24–26], DIANA microT version 3 (http://diana.cslab.ece.ntua.gr/microT/) [27], [28], miRDB version 4 (mirdb.org/) [29], [30], miRWalk March 2011 update (www.ebi.ac.uk/enright-srv/microcosm/htdocs/targets/v5/) [31], PITA 2007 release (genie.weizmann.ac.il/pubs/mir07/mir07_prediction.html) [32], RepTar version 1.2 (reptar.ekmd.huji.ac.il/) [33] and StarBase version 2 (starbase.sysu.edu.cn/) [34], [35]. The binding energy of between the 3’UTR and the miRNA was determined using available software RNA22 version 2 (https://cm.jefferson.edu/rna22/Interactive/) [36].

### *In silico* analysis

Bioinformatics prediction of miRNAs targeting CD46 was performed using available databases online. Pathway analysis was conducted using DIANA miRPath version 2 (http://diana.imis.athena-innovation.gr/DianaTools/index.php?r=mirpath/index) [37]. Gene ontology (GO) analysis was performed using GeneCodis version 3 (http://genecodis.cnb.csic.es/) [38–40], using the list of predicted genes from miRPath.

### Statistical and data analysis

Statistical significance was set at *p-value <0*.*05*. All analyses were performed using Microsoft Excel 2010 and SPSS version 16. MiRNA expression levels were expressed as fold change relative to the respective control samples. Hierarchical clustering was plotted using TIGR Multiple Experiment Viewer (TMeV; http://www.tm4.org/mev/) [41]

## Results

### *CD46* mRNA expression in various stroke subtypes

**Fig 1:**
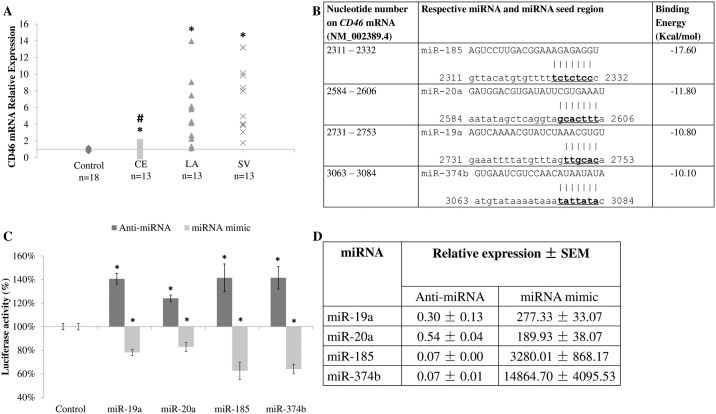
*CD46* mRNA level in cardioembolic and non-cardioembolic (large artery and small vessel stroke) patients and miRNAs targeting *CD46*. **A**. *CD46* mRNA level in patients. Data expressed as relative expression with respect to control samples, with the number of patients and controls indicated. **CE** represents cardioembolic stroke. **LA** represents large artery stroke. **SV** represents small vessel stroke. Data in **A** are shown as relative expression. **B**. miRNA binding sites and binding energies. Nucleotides in bold denoted the seed region. **C**. Luciferase assay. **D**. miRNA levels in transfected HeLa cells. Data in **C** and **D** are shown as mean ± SD. All experiments were performed in n = 3. * denotes significant difference (*p* < 0.05) against control (**A** and **C**) using Mann-Whitney U test. # denotes significance between cardioembolic and non cardioembolic stroke (**A**) using Mann-Whitney U test.

*CD46* mRNA measurements were quantitated in ischemic stroke patients (Patients’ demography in Table 1). *CD46* mRNA levels were found to be significantly higher (*p-value* < 0.001) in large artery (median = 5.05; interquartile range = 2.54–6.46) and small vessel stroke patients (median = 6.45; interquartile range = 3.87–9.90) as compared with cardioembolic stroke patients (median = 1.32; interquartile range = 0.86–1.58; Fig 1A). This observation is consistent with previous findings that *CD46* mRNA was differentially expressed between cardioembolic stroke and non-cardioembolic stroke (large artery and small vessel stroke) [6], [7]. Thus, it is important to identify the miRNAs that target *CD46* mRNA in order to demonstrate the potential of using miRNAs as biomarkers to distinguish cardioembolic stroke from non-cardioembolic stroke.

### Identification of miRNAs targeting *CD46* mRNA

Bioinformatics analysis was performed to identify miRNAs targeting *CD46* mRNA. A total of 9 miRNA prediction databases that were available online were used [17–36]. A total of 857 miRNAs (at least in one of the database) have been found to target CD46 3’UTR. This was narrowed down to 157 miRNAs by comparison with our group’s previously published data on miRNA profiles from ischemic stroke patients [42]. Among them, 12 miRNAs (miR-17, -18a, -19a, -19b, -20a, -20b, -106b, -181a, -181b, -185, -374b and -421) were predicted by 5 or more databases to target *CD46* mRNA. Of these, miR-19a, -20a, -185 and -374b were finally selected as they had the lowest binding energies to the *CD46* 3’UTR (Fig 1B).

### miR-19a, -20a, -185 and -374b targets *CD46* mRNA

In order to validate that miR-19a, -20a, -185 and -374b target *CD46* mRNA, luciferase reporter assay was performed. The 3’ untranslated region (3’UTR) of CD46 was sub-cloned into the pMIR-REPORT^™^ plasmid and used for co-transfection with respective anti-miRNA or miRNA mimics in HeLa cells. The success of transfection was verified by measuring the level of respective miRNAs in the transfected cells (Fig 1C). Cells transfected with anti-miRNAs exhibited an increase in relative luciferase activity, while cells transfected with miRNA mimics showed a reduction in relative luciferase activity (Fig 1D). The results demonstrated that *CD46* mRNA is a *bona fide* target of miR-19a, -20a, -185 and -374b.

### Differential miRNA expression between cardioembolic stroke and non-cardioembolic stroke subtypes

**Fig 2:**
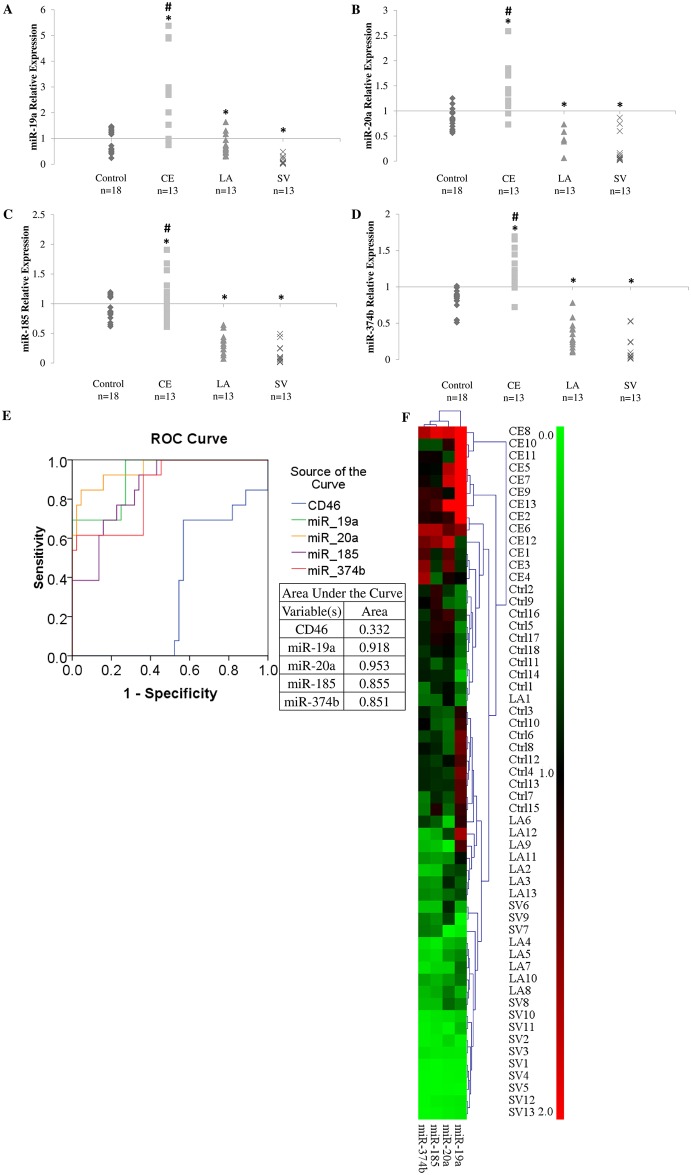
miRNAs level in cardioembolic and non-cardioembolic (large artery and small vessel stroke) patients. **A**. miR-19a. **B**. miR-20a. **C**. miR-185. **D**. miR-374b. * denotes tested to be significantly different (*p* < 0.05) from control (**A**—**D**) using Mann-Whitney U test. # denotes tested to be statistically significant between cardioembolic and non cardioembolic stroke. **E**. Receiver operating characteristic (ROC) curve for *CD46*, miR-19a, -20a, -185 and -374b. **F**. Hierarchical clustering of control and patients according to miRNA levels. CE represents cardioembolic stroke. LA represents large artery stroke. SV represents small vessel stroke. Ctrl represents control. Data are shown as relative expression. Red represents up-regulation. Green represents down-regulation.

Our investigation showed that the expression of miR-19a, -20a, -185 and -374b were significantly reduced in large-artery stroke (Median: miR-19a = 0.63, miR-20a = 0.42, miR-185 = 0.32, miR-374b = 0.27; Interquartile range: miR-19a = 0.47–0.94, miR-20a = 0.26–0.68, miR-185 = 0.23–0.39, miR-374b = 0.20–0.42; Fig 2A–2D) and small vessel patients (Median: miR-19a = 0.07, miR-20a = 0.06, miR-185 = 0.07, miR-374b = 0.05; Interquartile range: miR-19a = 0.03–0.19, miR-20a = 0.03–0.16, miR-185 = 0.02–0.24, miR-374b = 0.01–0.23; Fig 2A–2D) when compared with the cardioembolic stroke patients (Median: miR-19a = 2.69, miR-20a = 1.36, miR-185 = 1.05, miR-374b = 1.23; Interquartile range: miR-19a = 0.98–2.98, miR-20a = 1.17–1.80, miR-185 = 0.85–1.30, miR-374b = 1.06–1.53; Fig 2A–2D). This is as expected based on our observation that the expression of *CD46* was higher in the non-cardioembolic stroke patients, and thus strongly supports our earlier findings that these miRNAs target *CD46* mRNA.

Furthermore, using the expression of *CD46* and the expression of miRNAs targeting *CD46* (miR-19a, -20a, -185 and -374b), a receiver operating characteristic (ROC) curve was obtained (Fig 2E). The plot shows that the miRNAs were more effective in distinguishing cardioembolic stroke patients from non-cardioembolic stroke patients and healthy controls (Fig 2E). This was denoted by the larger area under curve (AUC) value for the miRNAs than *CD46* (AUC value: *CD46* = 0.332, miR-19a = 0.918, miR-20a = 0.953, miR-185 = 0.855, miR-374b = 0.851). Using TMeV [41], the miRNA expression can be used to plot for hierarchical clustering, which showed segregation of cardioembolic stroke samples from controls and noncardioembolic stroke samples (Fig 2F). Hence, these miRNAs may prove to be useful biomarkers for cardioembolic stroke diagnosis.

### *In silico* analysis of pathophysiological mechanism of miRNAs targeting *CD46*

In order to determine the possible mechanism behind the dysregulation of *CD46* and its respective miRNAs, *in silico* analysis was performed to decipher the potential pathways and processes that were affected during the dysregulation. Pathway analysis was performed using DIANA miRPath version 2 [37]. The results showed that among the top 10 dysregulated pathways (based on enrichment score), there were 3 pathways (Wnt signalling pathway, TGF-beta signalling pathway and Gap junction) associated with endothelial cells (Table 2) [43–45]. Further investigation was conducted by focussing at biological processes and gene ontology (GO). GO analysis was performed on GeneCodis version 3 (Table 2) [38–40]. There were multiple biological processes associated with endothelial cells from the GO analysis, pointing to endothelial cells as the possible site of action for the dysregulated gene and miRNAs. Hence, further verification was performed with human umbilical vein endothelial cells (HUVECs) as a model.

**Table 2:** Table of pathways and Gene Ontology (GO) biological processes associated with miR-19a, -20a, -185 and -374b.

KEGG Pathway	*Enrichment Score*	GO Biological Process	*Enrichment Score*
Axon guidance	*24*.*08*	cell adhesion	*22*.*90*
***Wnt signaling pathway***	***23*.*66***	blood coagulation	*14*.*72*
Pathways in cancer	*23*.*50*	angiogenesis	*13*.*83*
p53 signaling pathway	*21*.*19*	canonical Wnt receptor signaling pathway	*8*.*99*
Ubiquitin mediated proteolysis	*20*.*44*	platelet activation	*7*.*46*
Transcriptional misregulation in cancer	*20*.*10*	wound healing	*7*.*40*
***TGF-beta signaling pathway***	***20*.*08***	positive regulation of angiogenesis	*5*.*74*
Endocytosis	*19*.*75*	Wnt receptor signaling pathway, calcium modulating pathway	*5*.*57*
Melanogenesis	*18*.*83*	vascular endothelial growth factor receptor signaling pathway	*5*.*44*
***Gap junction***	***17*.*13***	negative regulation of canonical Wnt receptor signaling pathway	*5*.*03*
GnRH signaling pathway	*16*.*65*	Wnt receptor signaling pathway	*4*.*55*
Prostate cancer	*15*.*97*	Notch signaling pathway	*4*.*51*
MAPK signaling pathway	*15*.*18*	cell-matrix adhesion	*4*.*35*
HTLV-I infection	*15*.*18*	negative regulation of cell adhesion	*3*.*95*
Salivary secretion	*13*.*52*	transforming growth factor beta receptor signaling pathway	*3*.*95*
Calcium signaling pathway	*13*.*35*	regulation of cell adhesion	*3*.*65*
ABC transporters	*13*.*09*	positive regulation of endothelial cell migration	*3*.*60*
Melanoma	*12*.*52*	positive regulation of vasoconstriction	*3*.*60*
Regulation of actin cytoskeleton	*12*.*17*	positive regulation of endothelial cell proliferation	*3*.*37*
ErbB signaling pathway	*12*.*17*	blood vessel development	*3*.*17*

### miR-19a and -20a regulate *CD46* expression in HUVECs

**Fig 3:**
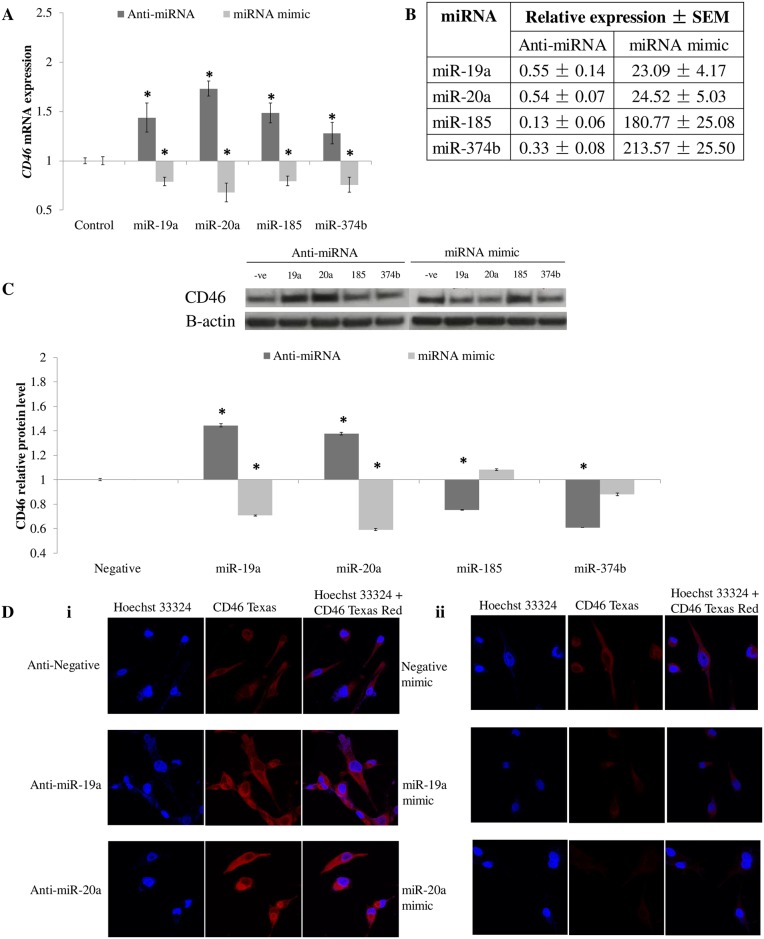
Anti-miRNA and miRNA mimic transfection in HUVECs. **A**. *CD46* mRNA expression in transfected HUVECs. **B**. miRNA levels in transfected HUVECs. **C**. Western blot analysis and respective quatification of CD46 protein in transfected HUVECs.–ve: Negative; 19a: miR-19a; 20a: miR-20a; 185: miR-185; 374b: miR-374b. **D**. Immunocytochemistry of transfected HUVECs. All experiments were performed in n = 3. * denoted tested to be significantly different (*p* < 0.05) from control (**A** and **C**) using Mann-Whitney U test. Data in **A** and **C** were shown as mean ± SD, n = 3.

In order to show miRNAs target *CD46* mRNA in endothelial cells, HUVECs were transfected with either anti-miRNA or miRNA mimics. *CD46* mRNA and protein were quantitated in these cells to determine the effect of miRNAs on *CD46* endogenously (Fig 3). The success of transfection was also verified by measuring the level of respective miRNAs in the transfected cells (Fig 3B). As expected, the transfection of anti-miRNA caused an increase in *CD46* mRNA level (anti-miR-19a = 1.43 ± 0.14, anti-miR-20a = 1.73 ± 0.07, anti-miR-185 = 1.48 ± 0.09, anti-miR-374b = 1.27 ± 0.10) while the introduction of miRNA mimic reduced the level of *CD46* mRNA (miR-19a mimic = 0.79 ± 0.04, miR-20a mimic = 0.67 ± 0.09, miR-185 mimic = 0.79 ± 0.04, miR-374b mimic = 0.75 ± 0.07; Fig 3A).

However, at the protein level, only miR-19a and -20a were shown to bring about an effect on CD46 protein level, which was quantified based on intensity of bands in Western blotting and measured using ImageJ software [16] The anti-miRNA transfected HUVECs had a significant increase in CD46 protein expression (anti-miR-19a = 1.44 ± 0.01, anti-miR-20a = 1.37 ± 0.00, anti-miR-185 = 0.75 ± 0.00, anti-miR-374b = 0.60 ± 0.00; Fig 3C; S1 Fig) while miRNA mimic transfection brought about a decrease in CD46 protein level (miR-19a mimic = 0.70 ± 0.00, miR-20a mimic = 0.59 ± 0.01, miR-185 mimic = 1.08 ± 0.00, miR-374b mimic = 0.87 ± 0.01; Fig 3C; S1 Fig). This was further verified through immunocytochemistry (Fig 3D). HUVECs transfected with anti-miRNAs (miR-19a and miR-20a) showed an increase in CD46 protein, denoted by the increase in red fluorescence as compared with control (Fig 3Di), whilst HUVECs transfected with miRNA mimics showed reduced red fluorescence (Fig 3Dii), signifying decrease in CD46 protein. These results demonstrated the relevance of miR-19a and -20a targeting *CD46* in endothelial cells.

## Discussion

Cardioembolic stroke is a subtype of ischemic stroke which is well-known for its high mortality and morbidity rate. Therefore, it is imperative to identify more accurate markers for cardioembolic stroke as it will have implications on the timely selection of appropriate therapy.

Jickling *et al* [6], [7] demonstrated that *CD46* mRNA was among a list of 40 mRNA as potential markers for distinguishing ischemic stroke subtypes. Among these 40 mRNAs, *CD46* seems to have a direct involvement in the pathophysiology of different ischemic stroke subtypes. This is not surprising as *CD46* is part of the complement cascade which regulates inflammation, a crucial pathological process in the pathophysiology of ischemic stroke. In this study, we have shown that *CD46* mRNA level to be significantly up-regulated in non-cardioembolic stroke while in cardioembolic stroke the *CD46* mRNA level appears to be similar to that of the healthy controls. This observation is in line with the current literature which suggests that atherosclerosis, the underlying pathology of large artery and small vessel stroke, is largely driven by the process of inflammation [46].

Furthermore, we have identified endothelial cells as a possible location. It is noteworthy to mention that endothelial cell dysfunction is an important hallmark in atherosclerosis and it was previously reported that CD46 protein level was found to be up-regulated in atherosclerotic plaque [47], [48]. Hence, the up-regulation of *CD46* mRNA found in patients with large artery and small vessel stroke is entirely in agreement with current evidence, signifying the crucial role that *CD46* plays in the pathogenesis of large artery and small vessel stroke.

In addition, by using *in silico* prediction and biochemical methodology we have identified miR-19a, -20a, -185 and -374b to be targeting *CD46*. We have utilized luciferase assay, a direct method that is used to prove the binding between miRNA:mRNA pair in various complementary binding studies [49], [50]. However, when CD46 protein expression were measured, only miR-19a and miR-20a proved to be useful to significantly modulate CD46 in HUVECs. miR-19a has been reported to protect endothelial cells from lipopolysaccharide-induced apoptosis, which is a characteristic of atherosclerosis [51]. The down-regulation of miR-19a detected in large artery and small vessel stroke implies the increase in lipopolysaccharide-induced apoptosis leading to ischemic stroke onset. This demonstrates the potential mechanism of miR-19a and -20a in endothelial cells during atherosclerosis and even possibly contributing to large artery and small vessel stroke.

During the pathway analysis, complement cascade was highlighted to be regulated by miR-19a, -20a, -185 and -374b. The complement cascade also interacts with the coagulation cascade. Coagulation cascade plays an important role in the formation of blood clot, the underlying cause for most ischemic stroke cases. There is a subtle difference in the induction of coagulation cascade between cardioembolic stroke and non-cardioembolic stroke (large artery and small vessel strokes), which is emphasized in the different treatments between cardioembolic stroke and non-cardioembolic stroke [52], [53]. Non-cardioembolic strokes are treated with anti-platelet drugs as the process of blood clotting is most likely induced *via* the tissue factor pathway due to tissue damage, such as rupture of the atherosclerotic plaque [52]. On the other hand, cardioembolic stroke is treated with anti-coagulants, suggesting that the blood coagulation follows the intrinsic pathway associated with pooling of blood, which corresponds to the pathogenesis of blood clot in cardioembolic stroke [53]. It is noteworthy to mention that miR-19a was reported to possibly modulate tissue factor pathway inhibitor (TFPI) which could signify that the up-regulation of miR-19a in cardioembolic stroke led to a pro-coagulation state in cardioembolic stroke patients [54]. Also, miR-20a was reported in a panel of miRNAs that could distinguish etiology of various ischemic stroke subtypes [37]. This lends further support to the relevance of this miRNAs in the pathogenesis of cardioembolic stroke.

Interestingly, results from the GO analysis showed that there were biological processes involved in coagulation cascade (blood coagulation and platelet activation). This seems to suggest that the deregulation of the complement cascade, involving *CD46* and miRNAs targeting *CD46* (miR-19a, -20a, -185 and -374b) may be potential markers for the differential regulation of coagulation cascade between cardioembolic stroke and non-cardioembolic stroke. A preliminary *in silico* prediction was performed to identify potential mRNA targets of miR-19a, -20a, -185 and -374b within the complement and coagulation cascade. It is noteworthy to mention that these miRNAs target several other genes in the pathways (miR-19a: 9 targets; miR-20a: 2 targets; miR-185: 6 targets; miR-374b: 5 targets; Table 3). Hence, a further investigation into miR-19a, -20a, -185 and -374b modulation of complement and coagulation cascade in blood cells may provide further understanding of the molecular mechanism behind the difference in complement and coagulation cascade activities between cardioembolic stroke and non-cardioembolic stroke.

**Table 3:** Predicted targets of miR-19a, -20a, -185 and -374b in complement and coagulation cascade.

hsa-miRNA	Predicted targets
miR-19a	*AT3*, ***CD46***, *CD55*, *F5*, *F13*, *PAI-1*, *TF*, *TFPI* and *PLAU*
miR-20a	***CD46*** and *PAI-1*
miR-185	***CD46***, *F7*, *F8*, *F11*, *F13* and *PLAT*
miR-374b	***CD46***, *CD55*, *F5*, *PAI-2* and *TFPI*

*AT3*: Anti-thrombin III. *CD46*: Cluster of differentiation 46. *CD55*: Cluster of differentiation 55. *F5*: Factor V. *F7*: Factor VII. *F8*: Factor VIII. *F11*: Factor XI. *F13*: Factor XIII. *PAI-1*: Plasminogen activator inhibitor-1. *PAI-2*: Plasminogen activator inhibitor-2. *PLAT*: tissue plasminogen activator. *PLAU*: urokinase-type plasminogen activator. *TF*: Tissue factor. *TFPI*: Tissue factor pathway inhibitor.

In the design of this study, *GAPDH* was used as an endogenous control for normalization of miRNA expression. The level of *GAPDH* was found to be consistent throughout all the experimental conditions. Despite the length of *GAPDH* mRNA being longer than the length of miRNA, numerous studies had used *GAPDH* as endogenous control for normalizing the level of miRNAs [55–59], which vindicates its role as an endogenous control for miRNA level normalization.

In brief, we have verified that *CD46* mRNA is differentially expressed between cardioembolic stroke and non-cardioembolic stroke subtypes, where it was up-regulated in non-cardioembolic stroke. Furthermore, miR-19, -20a, -185 and -374b have been demonstrated to target *CD46* mRNA and these miRNAs also showed correspondingly inversed expression when compared to the expression of *CD46* mRNA where the expression of the miRNAs were down-regulated in non-cardioembolic stroke. In addition, endothelial cells in non-cardioembolic stroke were likely to be affected by this dysregulation. Among the miRNAs, miR-19a and -20a are the most likely candidates to be involved in up-regulating CD46 through their down-regulation in endothelial cells. Based on the ROC plot, miRNAs were more accurate in the diagnosis of cardioembolic stroke patients where miR-19a and -20a have been found to have stronger AUC values (miR-19a = 0.918, miR-20a = 0.953). Nonetheless, more data are needed to identify the threshold range of *CD46* mRNA, miR-19, -20a, -185 and -374b for accurate diagnosis in stroke patients.

## Supporting information

S1 Fig Western blot analysis and respective quantification of CD46 protein in transfected HUVECs. **A**. CD46. **B**. Beta-actin. 1. Anti-miR-374b. 2. Anti-miR-185. 3. Anti-miR-20a. 4. Anti-miR-19a. 5. Anti-Negative. 6. miR-374b mimic. 7. miR-185 mimic. 8. miR-20a mimic. 9. miR-19a mimic. 10. Negative mimic.
(TIFF)
