# Supplementary figures and images for: MicroRNAs regulating cluster of differentiation 46 (CD46) in cardioembolic and non-cardioembolic stroke
Source: PLoS One. 2017 Feb 15;12(2):e0172131. doi: 10.1371/journal.pone.0172131 (PMC5310775; doi:10.1371/journal.pone.0172131)

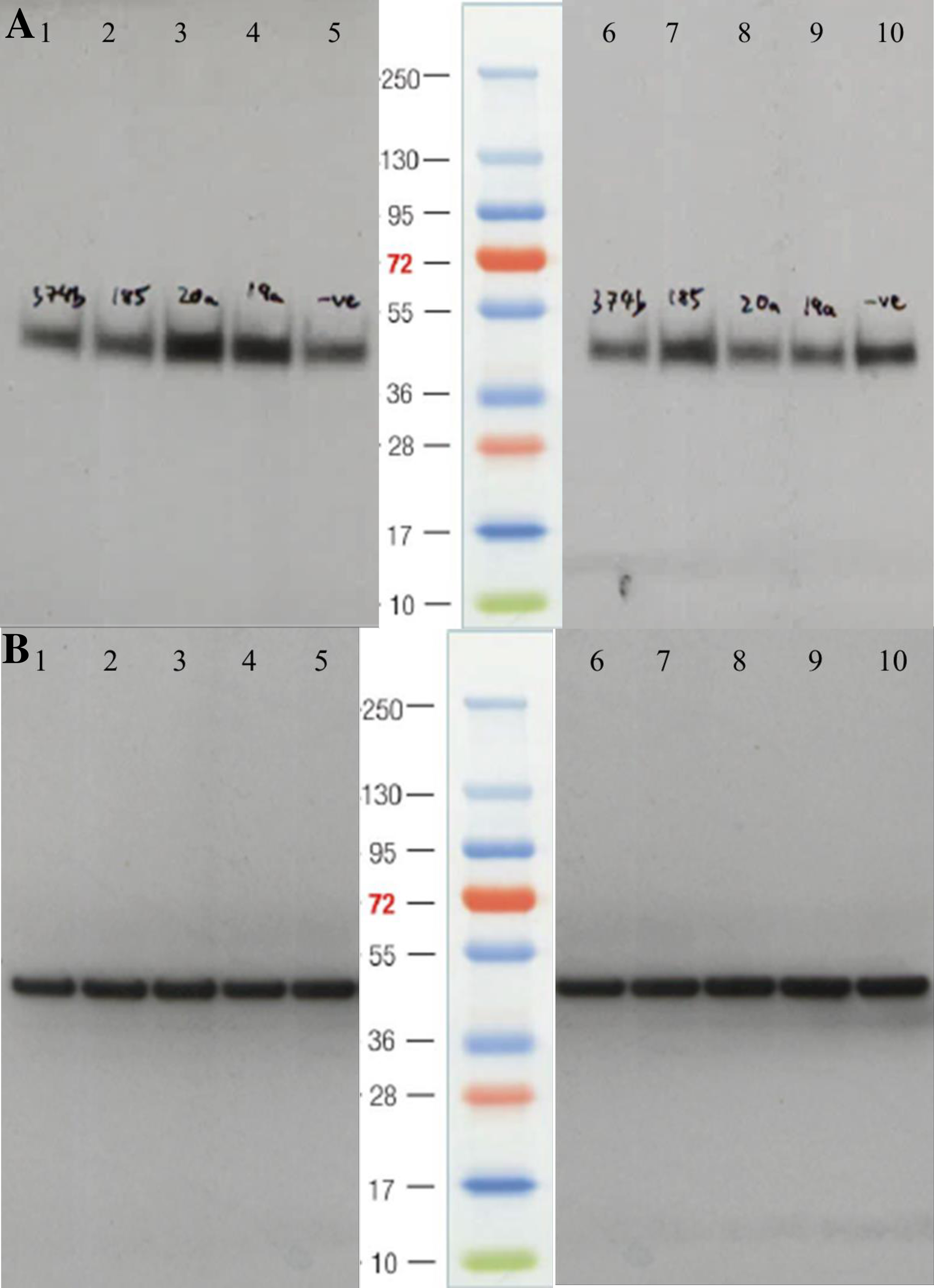

Supplement: S1 Fig — A. CD46. B. Beta-actin. 1. Anti-miR-374b. 2. Anti-miR-185. 3. Anti-miR-20a. 4. Anti-miR-19a. 5. Anti-Negative. 6. miR-374b mimic. 7. miR-185 mimic. 8. miR-20a mimic. 9. miR-19a mimic. 10. Negative mimic. (TIFF) [file pone.0172131.s001.tiff]
